# Supplementary figures and images for: G9a/GLP-sensitivity of H3K9me2 Demarcates Two Types of Genomic Compartments
Source: Genomics Proteomics Bioinformatics. 2020 Dec 5;18(4):359–70. doi: 10.1016/j.gpb.2020.08.001 (PMC8242262; doi:10.1016/j.gpb.2020.08.001)

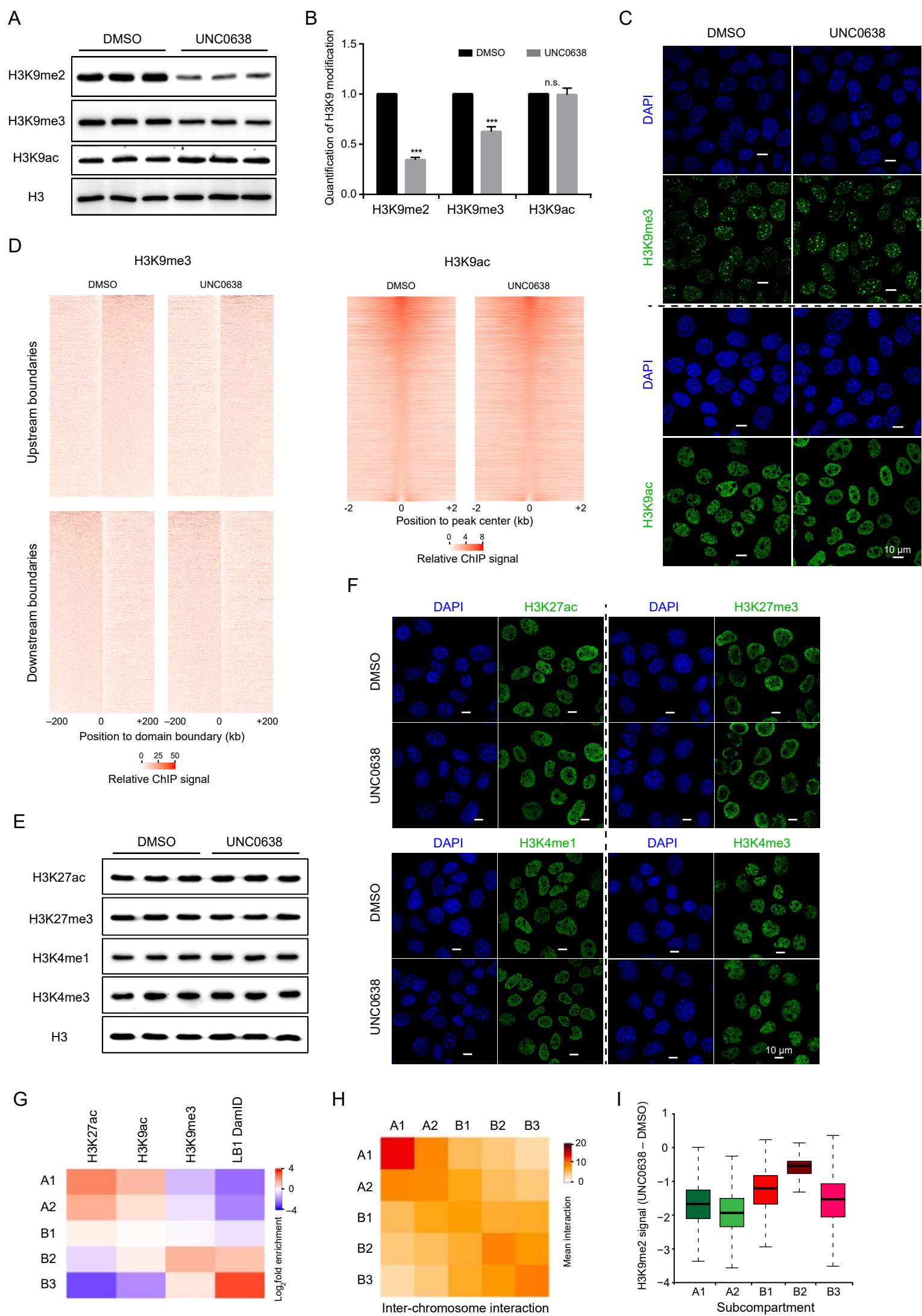

Supplement: Supplementary Figure S1 — Global levels of histone modifications upon UNC0638 treatment in AML12 cells. A. WB with antibodies against H3K9me2, H3K9me3, and H3K9ac in DMSO and UNC0638 treated AML12 cells, with three biological repeats respectively (independent DMSO/UNC0638 treatment). H3 serves as the loading control. WB, western blotting. B. Relative grayscale of WB from (A) calculated by AlphaView software. Student’s t-test. ***, P < 0.001; n.s., no significance. C. Representative IF images of H3K9me3 and H3K9ac in DMSO- and UNC0638-treated AML12 cells. Scale bar, 10 μm. D. Genome-wide alignments of H3K9me3 (left) and H3K9ac (right) ChIP-seq in DMSO and UNC0638 treated AML12 cells. The center of H3K9me3 heatmap is the boundary of its domain, and the center of H3K9ac heatmap is the called peak center. E. WB with antibodies against H3K27ac, H3K27me3, H3K4me1, and H3K4me3 in DMSO and UNC0638 treated AML12 cells, with three biological repeats respectively (independent DMSO/UNC0638 treatment). H3 serves as the loading control. F. Representative IF images of H3K27ac, H3K27me3, H3K4me1, and H3K4me3 in DMSO and UNC0638 treated AML12 cells. Scale bar, 10 μm. G. Heatmap showing the Log2 fold enrichment of H3K27ac, H3K9ac, H3K9me3, and LB1 DamID among five different subcompartments. H. Contact enrichment among the five subcompartments. I. Box plots showing the relative H3K9me2 levels (UNC0638 − DMSO) in subcompartments after UNC0638 treatment in AML12 cells. [file mmc1.pdf]

A

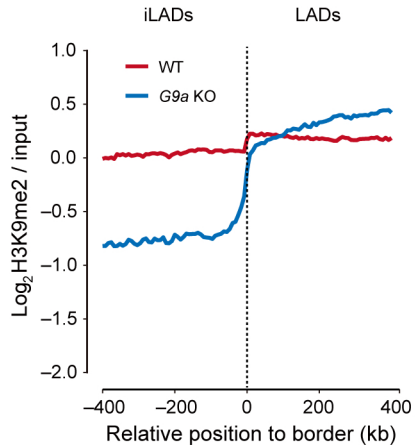

B

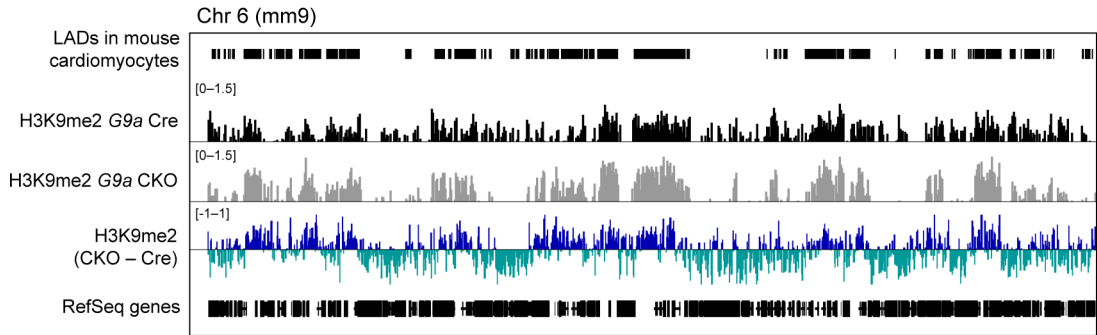

Supplement: Supplementary Figure S2 — Region-specific removal of H3K9me2 in G9a-KO MEF cells and G9a-CKO cardiomyocytes of mouse.A. Aligned H3K9me2 profiles of mirrored border regions of LADs in WT and G9a knockout MEF cells. H3K9me2 ChIP-seq data were from Chen et al. [27]. B. Representative tracks of normalized H3K9me2 ChIP-seq in Cre or G9a-CKO cardiomyocytes. Data were from Papait et al. [28]. [file mmc2.pdf]

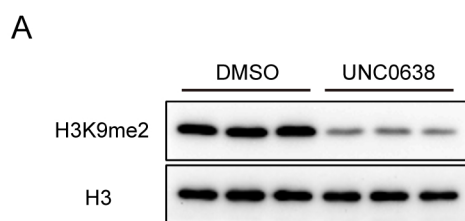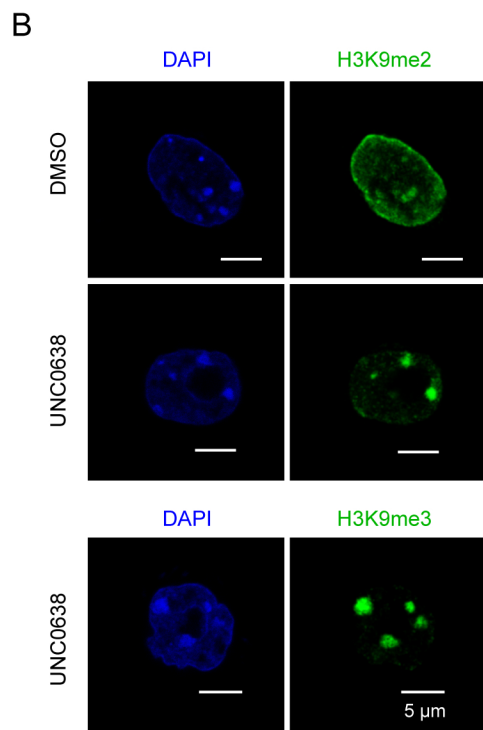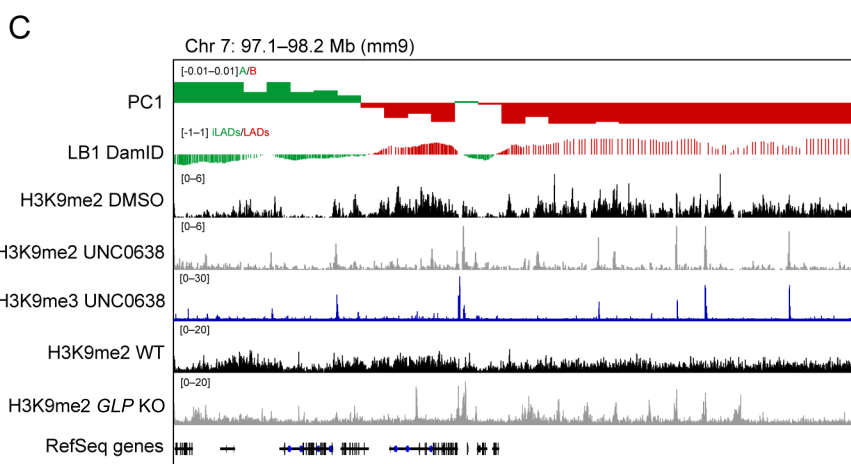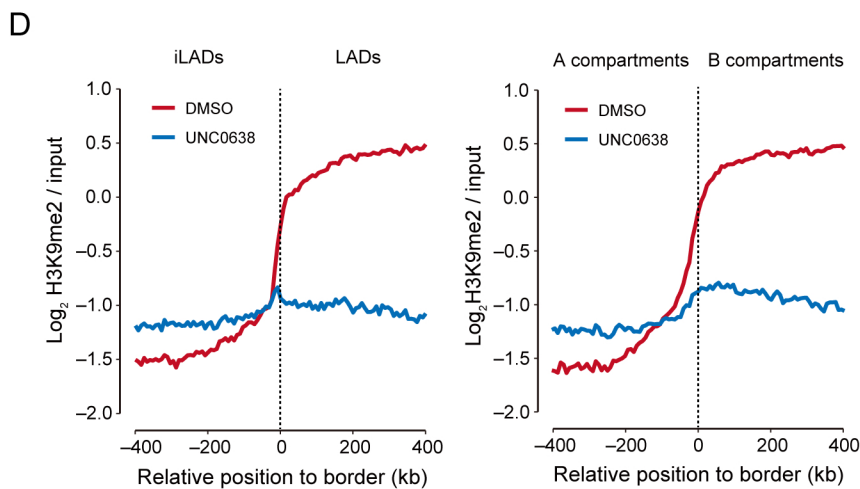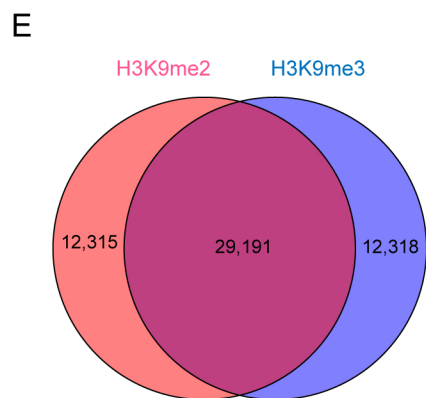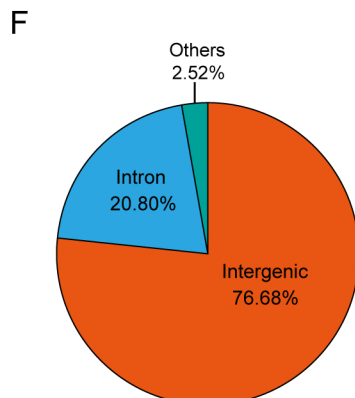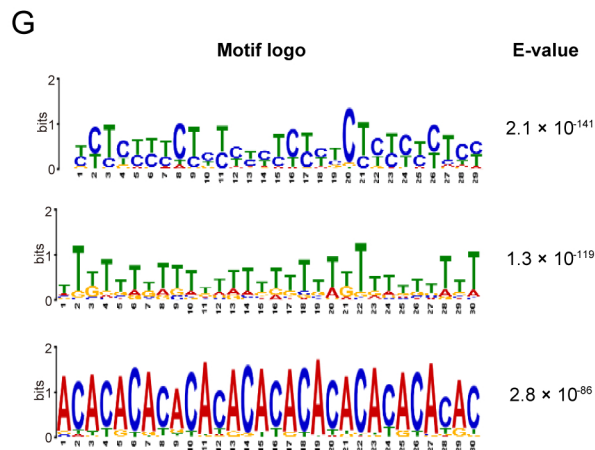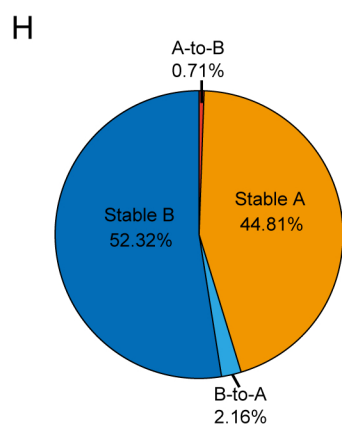

Supplement: Supplementary Figure S3 — Inhibition of G9a/GLP removes most of H3K9me2 modifications in mESCs. A. WB of H3K9me2 in DMSO and UNC0638 treated mESCs, with three biological repeats respectively (independent DMSO/UNC0638 treatment). H3 serves as the loading control. B. Representative IF images of H3K9me2 in DMSO and UNC0638 treated mESCs as well as H3K9me3 in UNC0638-treated mESCs. Scale bar, 5 μm. C. Representative H3K9me2 ChIP-seq tracks of DMSO/UNC0638 treated and WT/GLP KO mESCs, H3K9me3 ChIP-seq tracks of UNC0638 treated mESCs, and PC1 values and smoothed LB1 DamID signals of mESCs. D. Aligned H3K9me2 profiles of mirrored border regions of LADs (left) and A/B compartments (right) in DMSO- and UNC0638-treated mESCs. E. Venn diagram showing peaks overlapping between H3K9me2 and H3K9me3 ChIP-seq of UNC0638 treated mESCs. F. Proportions of the remaining H3K9me2 peaks not overlapped with H3K9me3 peaks in gene location of UNC0638 treated mESCs. G. Motif analysis of the remaining H3K9me2 peaks not overlapped with H3K9me3 peaks in UNC0638 treated mESCs. H. Compartment switching after UNC0638 treatment in mESCs. mESC, mouse embryonic stem cell. [file mmc3.pdf]
