# Supplementary material for: G9a/GLP-sensitivity of H3K9me2 Demarcates Two Types of Genomic Compartments
Source: Genomics Proteomics Bioinformatics. 2020 Dec 5;18(4):359–70. doi: 10.1016/j.gpb.2020.08.001 (PMC8242262; doi:10.1016/j.gpb.2020.08.001)

A

	DMSO-1	DMSO-2	UNC0638-1	UNC0638-2
Total reads	640,439,980	582,321,178	672,926,332	712,301,466
Side 1 aligned reads	598,512,108	533,521,356	629,798,007	652,237,504
Side 2 aligned reads	580,841,366	518,810,648	610,942,140	631,356,222
Unique pairs	421,058,021	371,234,965	451,181,870	460,807,465
Dangling ends pairs	14,988,485	16,588,610	17,686,126	35,586,488
Religation pairs	7,314,872	6,163,807	8,622,155	10,125,014
Self Cycle pairs	341,133	442,837	436,786	693,617
Dumped pairs	16,041	8,538	16,941	12,084
Duplicate pairs	133,826,010	62,113,717	145,789,620	83,005,005
Unique valid pairs	264,571,480	285,917,456	278,630,242	331,385,257
Unique valid pairs (%)	41.31	49.10	41.41	46.52
Cis interaction (< 20 kb)	36,488,503	39,202,818	40,863,418	46,106,198
Cis interaction (> 20 kb)	173,647,324	183,709,748	178,130,973	209,226,427
Trans interactions	54,435,653	63,004,890	59,635,851	76,052,632

B

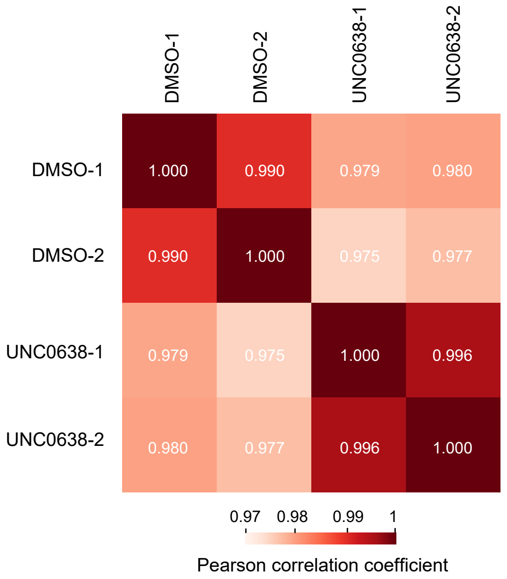

C

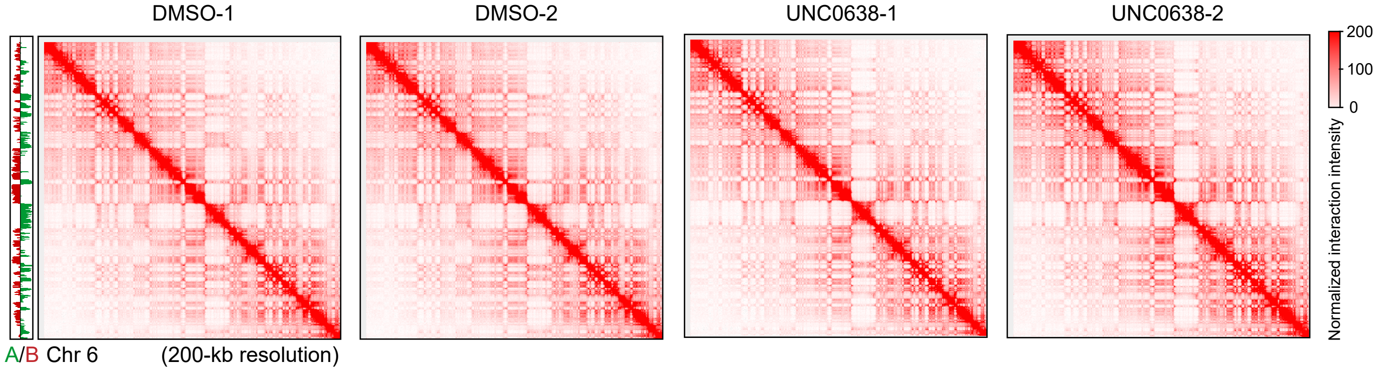

D

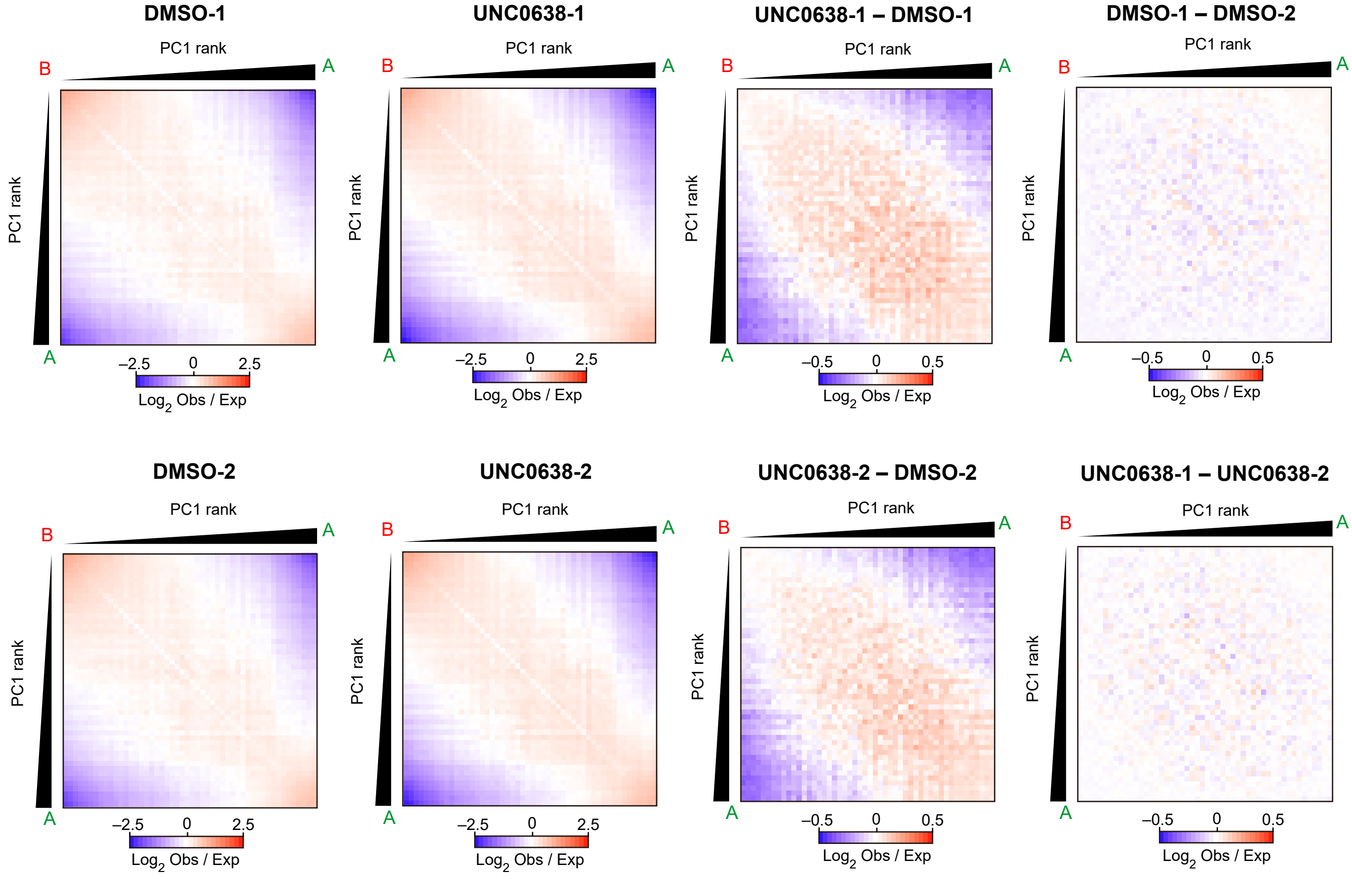

Supplement: Supplementary Figure S4 — Mapping statistics and reproducibility of Hi-C experiments. A. Mapping statistics of Hi-C deep sequencing data of DMSO and UNC0638 treated AML12 cells, with two biological repeats respectively (independent treatments, Hi-C assay and sequencing). B. Pearson correlation coefficients of PC1 values derived from compartment analysis between replicates. C. Representative contact matrices of DMSO and UNC0638 treated AML12 cells. D. Average contact enrichment between pairs of 250 kb bins ranked by PC1 values in DMSO (1st column) and UNC0638 (2nd column) treated AML12 cells, and the difference between them (3rd and 4th columns), with two biological repeats respectively. [file mmc4.pdf]
